# Ecological Determinants of Highly Pathogenic Avian Influenza (H5N1) Outbreaks in Bangladesh

**DOI:** 10.1371/journal.pone.0033938

**Published:** 2012-03-21

**Authors:** Syed S. U. Ahmed, Annette K. Ersbøll, Paritosh K. Biswas, Jens P. Christensen, Abu S. M. A. Hannan, Nils Toft

**Affiliations:** 1 Department of Large Animal Sciences, Faculty of Life Sciences, University of Copenhagen, Frederiksberg, Copenhagen, Denmark; 2 Department of Medicine and Surgery, Faculty of Veterinary Medicine, Chittagong Veterinary and Animal Sciences University, Chittagong, Bangladesh; 3 National Institute of Public Health, University of Southern Denmark, Copenhagen, Denmark; 4 Department of Microbiology, Faculty of Veterinary Medicine, Chittagong Veterinary and Animal Sciences University, Chittagong, Bangladesh; 5 Department of Veterinary Disease Biology, Faculty of Life Sciences, University of Copenhagen, Frederiksberg, Copenhagen, Denmark; 6 Epidemiology Unit, Department of Livestock Services, Dhaka, Bangladesh; U.S. Naval Medical Research Center Detachment, United States of America

## Abstract

**Background:**

The agro-ecology and poultry husbandry of the south Asian and south-east Asian countries share common features, however, with noticeable differences. Hence, the ecological determinants associated with risk of highly pathogenic avian influenza (HPAI-H5N1) outbreaks are expected to differ between Bangladesh and e.g., Thailand and Vietnam. The primary aim of the current study was to establish ecological determinants associated with the risk of HPAI-H5N1 outbreaks at subdistrict level in Bangladesh. The secondary aim was to explore the performance of two different statistical modeling approaches for unmeasured spatially correlated variation.

**Methodology/Principal Findings:**

An ecological study at subdistrict level in Bangladesh was performed with 138 subdistricts with HPAI-H5N1 outbreaks during 2007–2008, and 326 subdistricts with no outbreaks. The association between ecological determinants and HPAI-H5N1 outbreaks was examined using a generalized linear mixed model. Spatial clustering of the ecological data was modeled using 1) an intrinsic conditional autoregressive (ICAR) model at subdistrict level considering their first order neighbors, and 2) a multilevel (ML) model with subdistricts nested within districts. Ecological determinants significantly associated with risk of HPAI-H5N1 outbreaks at subdistrict level were migratory birds' staging areas, river network, household density, literacy rate, poultry density, live bird markets, and highway network. Predictive risk maps were derived based on the resulting models. The resulting models indicate that the ML model absorbed some of the covariate effect of the ICAR model because of the neighbor structure implied in the two different models.

**Conclusions/Significance:**

The study identified a new set of ecological determinants related to river networks, migratory birds' staging areas and literacy rate in addition to already known risk factors, and clarified that the generalized concept of free grazing duck and duck-rice cultivation interacted ecology are not significant determinants for Bangladesh. These findings will refine current understanding of the HPAI-H5N1 epidemiology in Bangladesh.

## Introduction

The emergence of an infectious disease combines two elements: the introduction of the pathogen in a susceptible population and its subsequent spread and maintenance within the population [1]. Ecological determinants can influence either or both of the elements and are recognize to have an important role in the emergence of diseases [1]. Recently, the role of ecological determinants favoring emergence and maintenance of avian influenza was discussed based on a proposed evolutionary ecological consideration [2]. The role of natural and artificial (human made) ecosystems and their components on the evolution of highly pathogenic avian influenza virus (HPAI), their adoption and persistence, have been previously described [2]. Recent studies have focused on environmental determinants contributing to the emergence and maintenance of HPAI subtype H5N1 (HPAI-H5N1) in several different countries, in order to clarify their role in HPAI-H5N1 epidemiology [3]–[7].

**Figure 1:**
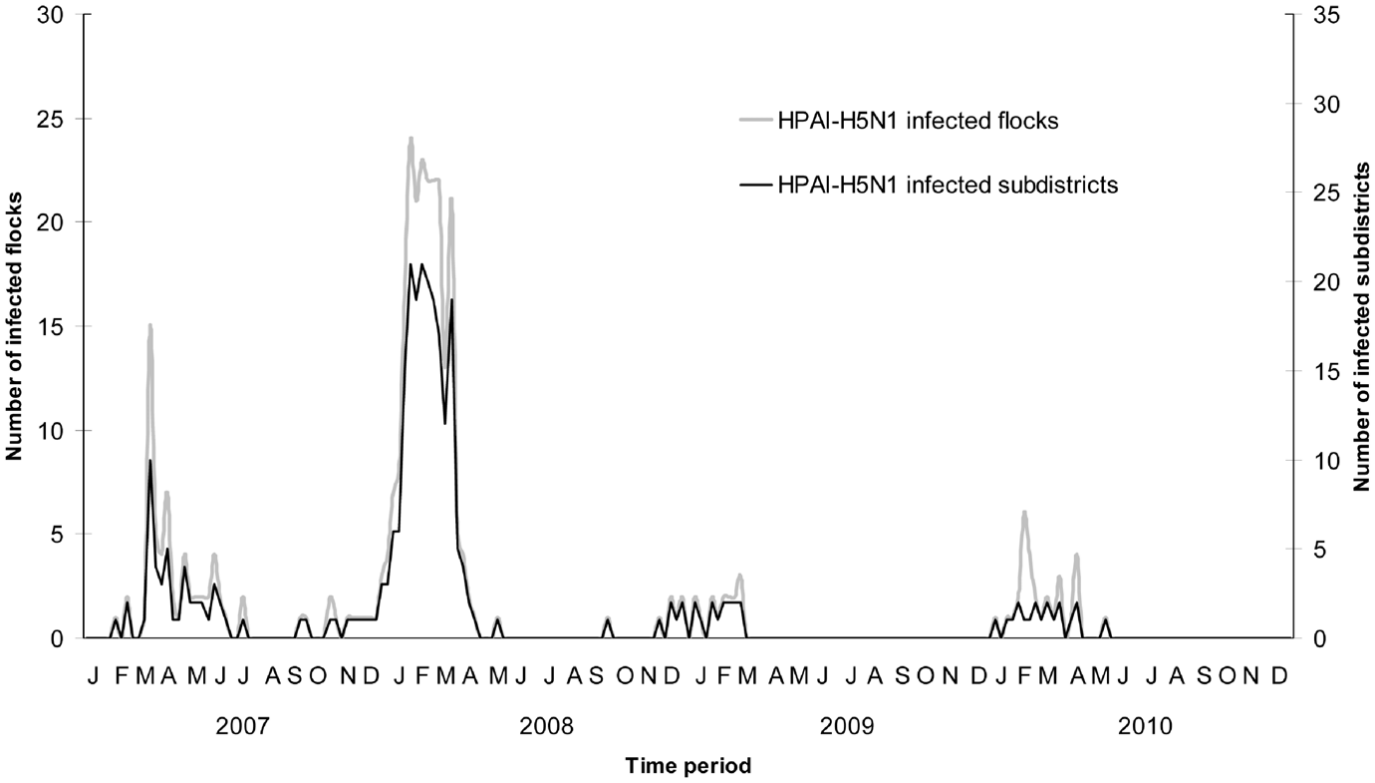
Temporal distribution of the weekly H5N1-HPAI virus outbreak records reported in Bangladesh.

Differences in the components of surveillance systems for HPAI-H5N1 in different countries complicate the comparison of outbreaks status of different countries. However, the number of outbreaks reported by Bangladesh from 2007 through 2010 placed the country among the highest reporting countries (http://www.oie.int/downld/AVIAN%20INFLUENZA/Graph%20HPAI/graphs%20HPAI%2005_12_2010.pdf). Although most countries succeeded in clearing the virus in the initial phase of the outbreaks, the virus has become endemic in poultry, in a number of affected countries including Bangladesh, Egypt, Indonesia and Vietnam [8]. Since the first outbreak in January 2007 till December 2010, Bangladesh faced four epidemic waves in four consecutive years (Figure 1), reported 370 flock level outbreaks in 148 subdistricts (n = 481) [9] and one human case [10]. Persistence of the virus and resurgence of the outbreaks every year in Bangladesh could complicate the epidemiology of HPAI-H5N1 because of very high human and poultry density [11].

The agro-ecology and poultry husbandry of the south and south-east Asian countries share common features, however, with noticeable differences between countries. The duck population of Bangladesh, Thailand and Vietnam is 38.1 [12] million, 69 million and 33 [13] million respectively with corresponding land area of 147,570 km^2^, 514,000 km^2^ and 313, 210 km^2^ [14]. Thus, overall duck density is higher in Bangladesh than Thailand and Vietnam. However, duck husbandry in Thailand and Vietnam is considerably more intensive [15] than Bangladesh. In Bangladesh, ducks are mostly reared for egg production as a source of household income and kept in traditional scavenging system without any additional input [15]. The ecology of duck production systems also varies between countries. For instance, the commercial duck production cycle in Thailand is closely connected with rice crop cultivation as rice is a source of feed for ducks [13], [16]. Furthermore, the movement of the commercial duck flocks is a common marketing practice in Thailand [13], [16], [17]. Both practices were significantly associated with risk of HPAI outbreaks in Thailand [3]. However, in Bangladesh commercial ducks husbandry is defined by the presence of large water bodies and flock movement of duck is very uncommon [12]. River transportation is a very important trade route in Bangladesh [18], which could also play an important role in the dispersion of the host and HPAI-H5N1 viruses within the country. River transportation as a contribution to HPAI-H5N1 epidemiology is not considered in the studies conducted in Thailand and Vietnam [4], [5], [19]. Live bird markets are considered to be neither practical nor popular in Thailand [5], whereas these are the single most important outlets for poultry and poultry meat in Bangladesh [12]. Hence, the determinants associated with the ecologic risk of HPAI-H5N1 in Bangladesh are expected to differ from Thailand and Vietnam.

A set of spatial determinants was identified in Bangladesh [11]. However, that study excluded natural ecological components, for example, any information regarding the migratory birds. An earlier study [20] indicated that the direction of the HPAI-H5N1outbreaks spread in Bangladesh was latitudinal (north to south or vice versa), and coincided with the Central Asian Flyways and East Asian Flyways. Migrant birds of different species use Bangladesh as their staging areas whilst following the Central Asian Flyways and East Asian Flyways. This links Bangladesh to China and Mongolia where HPAI-H5N1 outbreaks are predominant [20]. We believe that a more comprehensive study incorporating the analysis of migratory bird's staging areas, environmental determinants and more components of the poultry value chain (an organized system of exchange from production to consumption that increases the value of poultry) is required in order to obtain more accurate information concerning the ecological risk of experiencing HPAI-H5N1 in Bangladesh. Thus, we included migratory birds' staging areas, intersection of river networks, live bird markets, literacy rate, elevation and vegetation index, as additional variables along with population density, poultry density, duck density, road networks and crop intensity already analyzed in an earlier study [11].

Ecological studies, such as the present, are susceptible to clustering effects of spatial data. Data close together in space (e.g. neighboring subdistricts) are often more alike (correlated) than those are far apart [21]. This tendency is known as spatial autocorrelation (spatial clustering) and an ecological study must account for this. Griffith emphasized the importance of using a reasonable method for modeling spatial proximity, rather than assuming that the spatial data are independent [22]. He also emphasized the importance of the principle of parsimony; choose the simplest model that adequately explains the variation in the data and facilitates an interpretation, which is consistent with empirical knowledge [22]. Rasmussen proposed two generalized linear mixed modeling (GLMM) approaches that are able to address spatial clustering [23]. GLMMs have become the standard modeling approach for binary outcome from data with potential (spatial) clustering [24], [25], [26].

The primary aim of the current study was to quantify the ecological determinants associated with the risk of HPAI-H5N1 at subdistrict level in Bangladesh. Such information might provide valuable insights into targeting surveillance efforts in a country struggling to manage recurrent outbreaks with limited resources. The secondary aim was to explore the performance of two different statistical modeling approaches described by Rasmussen [23], taking into account the unmeasured spatially correlated variation.

## Materials and Methods

### Study population

Data on outbreaks of HPAI-H5N1 in Bangladesh were obtained from updates on HPAI in animals from the world Organization for Animal Health (OIE) that were officially reported by the Department of the Livestock services (DLS), Bangladesh [27]. The virus was confirmed in submitted tracheal samples by diagnostic laboratories, with the use of reverse transcriptase polymerase chain reaction [28]. The official report included the physical address including subdistrict name, detection date as well as geographical coordinates of the outbreak location. The outcome variable of this study was outbreak status at subdistrict level. If a subdistrict had at least one officially confirmed outbreak during the first and second epidemic wave, the subdistrict was considered as infected. All other subdistricts were considered as non-infected. We used the outbreak locations from third and fourth waves to assess the predictive power of the fitted models. Bangladesh is administratively divided into 481 subdistricts (mean area, 314 km^2^; median area, 271 km^2^; range, 36–1968 km^2^). Of the 481 subdistricts, we excluded 17 subdistricts due to the lack of data availability regarding ecological determinants and spatial structure resulting in a sample of 464 subdistricts. The spatial distribution of the infected subdistricts in first and second waves are shown elsewhere [29].

### Determinants

The ecological determinants assessed in this study were carefully chosen considering the plausibility of an association. The introduction, spread and persistence of the HPAI-H5N1 outbreaks are complex and probably multi-factorial, and all plausible determinants are not necessarily causal. We investigated determinants from both the natural and the artificial ecosystem. The determinants were poultry demographic characteristics, including host density; host dispersal determinants, like human density (proxy of human activity); migratory birds' staging areas; poultry value chain, including road transportation, river transportation and live bird markets; agro-ecology, e.g. crop intensity, elevation and vegetation index. An additional variable incorporated in this study was literacy rate to take account of potential reporting bias of the existing clinical symptom-based surveillance.

Data on poultry flock density, vegetation index, crop intensity and elevation were available and obtained from FAO GeoNetwork (http://www.fao.org/geonetwork/) in raster format. The highway and river networks were digitized based on Google map, and subsequently converted into a raster map. From the raster data, we summarized all variables at subdistrict level using ArcView Spatial Analyst's zonal statistics function. The household density and literacy rate were available at subdistrict level from Bangladesh population census. We used the number of economic centers (subdistrict headquarter, number of unions (lower administrative unit of subdistrict), and metropolitan wards (unit of metropolitan city)) as a proxy of live bird markets, also available from Bangladesh population census. Number of ducks per subdistrict was available from DLS. Data on the distribution of migratory bird's staging areas in Bangladesh (Figure S1) was obtained from the literature [30], [31], [32]. We used continuous and dichotomous variables, and where necessary some of the continuous variables were classified into plausible and appropriate categories using deciles classification [33]. All determinants used in the study are summarized with their possible categories and their importance in Table 1.

**Table 1:** Highly pathogenic avian influenza in Bangladesh, 2007–2008; classification and description of the independent variables included in the regression analysis.

Main category	Subcategory	Ecological determinants	Unit per subdistrict	Role in HPAI ecology	Plausible role in HPAI Epidemiology			
					I†	S‡	P¥	SRψ
Natural ecology	Migratory bird	Migratory birds' staging area	Present/Absent	Interface for spill over and spill back	X	X	X	X
	Environmental	Elevation	Meter above sea level	Host habitat				X
		Subdistrict in the main river network	Intersect subdistrict (Yes/No)	Host dispersal	X	X	X	X
		Vegetation index		Host habitat			X	X
Artificial ecology	Human demography	Household density	Houses per km^2^	Host dispersal	X	X		X
		Literacy rate	Percentage	Reporting bias				X
	Poultry demography	Poultry density	Number of poultry per km^2^	Host density		X	X	X
		Duck density	Number of ducks per km^2^	Host density	X	X	X	X
	Poultry value chain	National highway network	Intersect subdistrict (Yes/No)	Host dispersal	X	X		X
		Live bird market (LBM)	Number of LBM	Transmission interface	X	X	X	X
	Agro-ecology	Crop intensity	High/Medium/Low	Host habitat	X	X		X

^†^Introduction,

^‡^Spread,

¥ Persistance,

ψ Surveillance.

### Univariable analysis

All variables were individually tested for an association with the outcome status of subdistricts by univariable logistic analysis while taking into account the spatial correlation, using the GLIMMIX macro in SAS 9.2 (SAS Institute Inc., Cary, NC, USA). Variables that were statistically significant at p≤0.2 were included for further analysis. Regression coefficients were converted to odds ratio by exponentiation of their value and corresponding 95% CI.

### Multivariable analysis

Considering the spatial autocorrelation, we used generalized linear mixed models (GLMM) to take account of random effects for an area influenced by its neighbors, and thus, accounted for spatial autocorrelation. To assess the importance of the spatial autocorrelation, we compared two different modeling approaches. The first approach used an intrinsic conditional spatial autoregression (ICAR) with a traditional generalized linear mixed model [23]. The second approach used a parsimonious multilevel (ML) generalized linear mixed model [23], where we considered the random effect of the district for data on subdistricts nested within districts. Both models were conditional on random effects of the spatial neighborhood structure and based on restricted penalized quasi likelihood function (REPL). In the ICAR model, the spatial neighborhood of subdistricts was defined by Queens contiguity (subdistricts are neighbors if they share a common boundary or corner), and in the ML model, spatial neighborhood is defined by administratively occurring hierarchy (subdistricts nested within district). The difference between the definitions of neighborhood structure used in two different models is illustrated in Figure S2. For the comparison purpose, we fixed the dispersion parameter of the both ICAR and ML model to 1. We used GLIMMIX macro in SAS 9.2 (SAS Institute Inc., Cary, NC, USA) to run the ICAR model and ML model. Final models were fitted by using a backward elimination procedure. Colinearity was assessed by calculating the correlation between all covariates to be considered for inclusion in the final model. We tested two-way interactions and checked confounding among variables. Significance of determinants was assessed by using the likelihood ratio test based on p≤0.05. The deviances and Akaike's information criteria (AIC) for both ICAR and ML models were calculated and used for optimization. We converted regression coefficients of final models to odds ratios by exponentiation of the estimate and corresponding 95% CI.

### Predictive performance

The predictive performances of the models were assessed by the receiver operation characteristic (ROC) curve and area under the curve (AUC). Based on the predicted probability estimated by ICAR and ML models we generated predictive probability maps for Bangladesh. The locations of the outbreaks for wave three and four in Bangladesh were plotted into the predictive maps. Overlay analysis were performed in ArcGIS, to check the agreement of outbreak location with predicted high risk subdistricts by two different models.

## Results

### Study population

During the first two epidemic waves, 138 out of 464 subdistricts (29.7%) met the criteria to be classified as infected subdistricts and the remaining 326 (70.3%) were considered non-infected subdistricts.

### Univariable analysis

Results of the univariable logistic analyses are shown in Table 2. Nine of the eleven variables yielded p-values≤0.2 and were subsequently included in the multivariable analyses. The univariable analyses revealed that the subdistricts with migratory birds' staging areas, intersection of rivers and national highways, high household and poultry density, higher number of live bird markets and higher literacy rates were more likely to have outbreaks reported.

**Table 2:** Univariable logistic analysis of potential ecological determinants of HPAI-H5N1 virus outbreaks at subdistrict level in Bangladesh.

Ecological determinants	Unit per subdistrict	No. (%)of infected subdistrict (N = 138)	No. (%)of non-infected subdistricts (n = 326)	Estimate(SE)	OR (95% CI)	p
Migratory birds' staging area	Absent	119(86.2)	311(95.4)	0	1	0.001
	Present	19(13.8)	15(4.6)	1.197 (0.363)	3.310 (1.623–6.751)	
Elevation	Meter above sea level			−0.008 (0.005)	0.992 (0.983–1.002)	0.089
Subdistrict in the main river network (intersect subdistrict)	No	49(35.5)	158(48.5)	0	1	0.011
	Yes	89(64.5)	168(51.5)	0.535 (0.210)	1.708 (1.131–2.581)	
Vegetation index				0.204 (0.109)	1.227 (0.990–1.520)	0.063
Household density (houses per km^2^	≤135	11(8.0)	116(35.6)	0	1	<0.001
	135–270	91(65.9)	182(55.8)	1.663 (0.342)	5.273 (2.694–10.320)	
	>270	36(26.1)	28(8.6)	2.607 (0.405)	13.558 (6.117–30.055)	
Literacy rate (percentages)	≤50%	90(65.2)	277(83.4)	0	1	<0.001
	>50%	48(34.8)	54(16.6)	0.988 (0.233)	2.686 (1.699–4.248)	
Poultry density (number of poultry per km^2^)	≤700	21(15.2)	137(42.0)	0	1	<0.001
	700–1400	63(45.7)	137(42.0)	0.978 (0.293)	2.659 (1.495–4.726)	
	>1400	54(39.1)	52(16.0)	1.710 (0.305)	5.526 (3.035–10.063)	
Duck density (number of ducks per km^2^)	≤125	35(25.4)	99(30.4)	0	1	0.214
	125–250	44(31.9)	79(24.2)	0.455 (0.273)	1.575 (0.921–2.674)	
	>250	59(42.8)	148(45.4)	0.120 (0.251)	1.128 (0.689–1.845)	
National highway network (intersect subdistrict)	No	47(34.1)	207(63.5)	0	1	<0.001
	Yes	91(65.9)	119(36.5)	1.214 (0.214)	3.368 (2.213–5.126)	
Live bird market (LBM)	Number of LBM			0.075 (0.013)	1.078 (1.051–1.106)	<0.001
Crop intensity (percentage of arable land)	≤35%	15(10.9)	43(13.2)	0	1	0.432
	35–70%	35(25.4)	66(20.2)	0.419(0.367)	1.520 (0.739–3.125)	
	>70	88(63.8)	217(66.6)	0.151(0.327)	1.163 (0.612–2.208)	

### Multivariable analysis

The results of the multivariable models are shown in Table 3. Seven variables remained significant in the final ICAR model: subdistricts with a migratory bird area, intersection of the river and national highway, high household and poultry density, high number of live bird markets and high literacy rate were more likely to have outbreak reports than the other subdistricts. In the ML model, five variables remained significant: subdistricts with a migratory bird area, intersection of national highway, high household density, high number of live bird market and high literacy rate were more likely to have outbreak reports than the other subdistricts. The spatial autocorrelation in the ICAR model was insignificant but significant in ML model.

**Table 3:** Results of the multivariable analysis for ecological determinants, associated with risk of HPAI-H5N1 outbreaks at subdistrict level in Bangladesh.

		ICAR			ML		
Ecological determinants	Unit per subdistrict	Estimate (SE)	OR (95% CI)	Overall p	Estimate(SE)	OR (95% CI)	Overall p
Migratory birds' staging area	Absent	0	1	0.001	0	1	<0.001
	Present	1.820 (0.546)	6.173 (2.110–18.055)		1.995(0.560)	7.351 (2.444–22.111)	
Subdistrict in the main river network (intersect subdistrict)	No	0	1	0.045			
	Yes	0.524 (0.260)	1.688 (1.013–2.814)				
Household density (houses per km^2^)	≤135	0	1	0.003	0	1	0.002
	135–270	1.354 (0.429)	3.873 (1.665–9.009)		1.307(0.440)	3.695 (1.555–8.777)	
	>270	1.753 (0.528)	5.773 (2.046–16.290)		1.911(0.543)	6.759 (2.324–19.656)	
Literacy rate (percentages)	≤50%	0	1	0.002	0	1	0.026
	>50%	1.055 (0.337)	2.872 (1.479–5.577)		0.787(0.351)	2.197(1.101–4.382)	
Poultry density (number of poultry per km^2^)	≤700	0	1	0.048			
	700–1400	0.409 (0.366)	1.505 (0.733–3.091)				
	>1400	0.933 (0.397)	2.542 (1.163–5.551)				
National highway network (intersect subdistrict)	No	0	1	0.004	0	1	0.001
	Yes	0.745 (0.260)	2.106 (1.263–3.512)		0.964(0.285)	2.623 (1.498–4.595)	
Live bird market (LBM)	Number of LBM	0.057 (0.016)	1.059 (1.026–1.092)	<0.001	0.069(0.017)	1.071 (1.037–1.107)	<0. 001
Model statistics							
Σ^2^ spat		0.790(0.550)		0.151	1.368 (0.488)		0.003
Deviances		382.789			339.435		
−2 residual log likelihood		2272.400			2284.400		
AIC		2274.400			2286.400		

### Predictive performance

**Figure 2:**
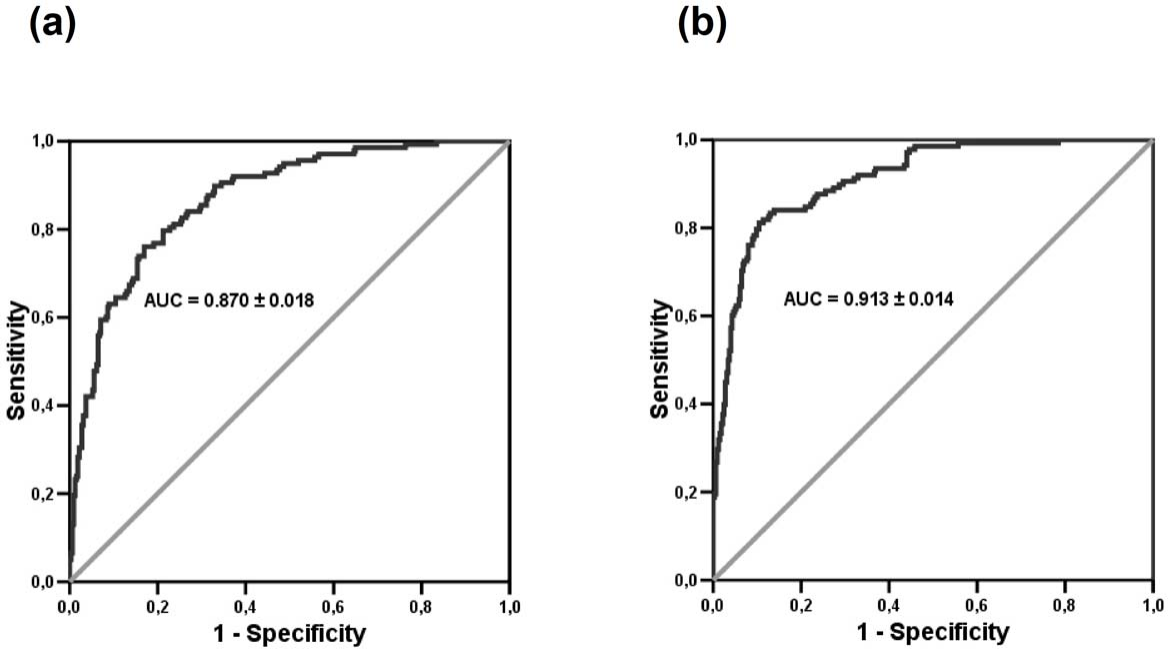
ROC curves of the predictive power of the (a) ICAR and (b) ML models.

**Figure 3:**
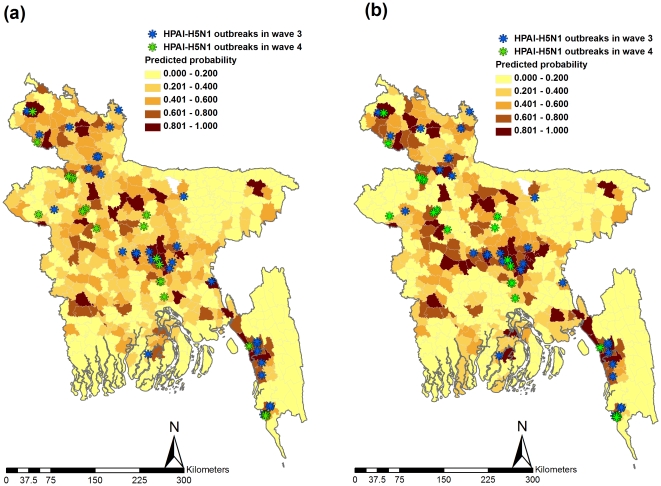
Predictive probability map from (a) ICAR and (b) ML models and overlay of the locations of H5N1-HPAI outbreaks in wave 3 and wave 4, in Bangladesh.

Both models have similar predictive performance (ICAR, AUC = 0.87±0.018; ML, AUC = 0.913±0.014) but are not identical. ROC curves, with AUC, for the predictive performance of both models, are illustrated in Figures 2a and 2b. Predictive probability maps of having a subdistrict infected based on ICAR and ML models were generated and presented (Figures 3a and 3b). Overlay analysis of outbreak locations for wave three on predicted probability map based on ICAR and ML revealed that 87.5% and 90.3%, of outbreaks overlaid on the moderate to high risk subdistricts, respectively. Outbreak locations in wave four overlaid 90.6% and 87.1% on the moderate to high risk subdistricts in predicted probability map based on ICAR and ML, respectively.

## Discussion

This ecological study examined and found additional ecological determinants associated with the HPAI-H5N1 outbreaks in Bangladesh compared to a previous study [11]. These new, significant, ecological determinants relate to migratory birds' staging areas, river networks, live bird markets and literacy rate. This new set of ecological determinants will refine current understanding of the HPAI-H5N1 outbreaks at subdistrict level of Bangladesh. The already known ecological determinants: poultry density, road networks and household density [11] were confirmed by the present study. The study also showed that the duck-rice cultivation interaction ecology for the perpetuation of HPAI-H5N1 in the south-east Asian countries [4], [13], [16] might not be an important factor in Bangladesh.

The current study demonstrates that the presence of migratory birds' staging area in a subdistrict increase the risk of having outbreaks of HPAI-H5N1 in that subdistrict, compared to a subdistrict without migratory birds' staging areas. According to the National Avian Influenza and Human Pandemic Influenza Preparedness and Response Plan for Bangladesh, 244 species of migratory birds' visit Bangladesh during winter. Of these species, at least 21 species may carry the HPAI-H5N1 [12]. These non-resident migratory wild birds use different wetlands, rivers and costal lines of Bangladesh for their resting and feeding and share these areas with different resident wild bird and poultry whilst they migrate through Bangladesh following Central Asian Flyways and East Asian Flyways. The migratory birds' staging areas and hotspots of HPAI-H5N1 outbreaks in Bangladesh coincide with east and central Asian flyways [34]. Thus, migratory birds might be important for the introduction and spread of HPAI-H5N1 in the poultry population of Bangladesh. These findings agree with those of previous studies in other countries [16], [35], [36]. The contact between poultry and wild birds is the most likely source of introduction of avian influenza virus [8]. As the densities of the human and livestock populations are very high in Bangladesh [11], the coexistence of the migratory birds' staging areas and human inhabitation in proximity is common. The proximity increases the chance of contact between poultry and wild birds. Backyard poultry sometimes share the habitat with migratory birds, which allows the infection to be introduced in the domestic poultry population [2]. As wild birds are not under the current surveillance, we strongly suggest monitoring the wild birds for the virus and further investigating the role of the wild birds in the introduction and reintroduction of HPAI-H5N1 in Bangladesh.

The river network is significantly contributing to the outbreaks at subdistrict level in Bangladesh. Subdistricts crossed by or at the banks of main river networks have an increased risk of HPAI-H5N1 outbreaks. In Bangladesh, river transportation is a significant trade route and an important component of the poultry value chain. The southern and south-eastern subdistricts are mostly connected to the capital by inland river transports [37]. Furthermore, small traders mostly use river networks and distribute their goods in the rural markets all over the country [18]. Infected poultry and contaminated poultry products and equipments transported through a river might promote spreads of HPAI-H5N1 in Bangladesh. Moreover, reports are available on the dumping of dead chickens [38]–[40] that contaminates the downstream environment [38]. Monitoring the poultry transportation by river transport and possible movement restrictions on birds from an infected zone to a non-infected zone need to be considered in Bangladesh. Biosecurity measures like quarantine of the birds coming from the infected zone, cleaning of the transportation material and disinfection programmed can be implemented in the river ports. Besides, the environmental hazard related to dumping of infected and dead chickens, needs to be assessed further.

Human behavior can influence the emergence of infectious diseases in a particular geographic location [1]. Understanding of the human behavior like interactions between humans and animals is very important to prevent and control infectious diseases [5]. The present study indicates that subdistricts with high household density had a higher incidence of outbreaks than those with a low density. This may reflect the fact that subdistricts with a high population density have larger poultry populations and poultry production related activities. High household density could also promote some risky human behavior for the dispersion of HPAI-H5N1. Human activities and movement are important for local transmission of the infection between flocks.

This study reveals that the risk of HPAI-H5N1 outbreaks in subdistricts increases with the poultry density in the subdistrict. Perhaps certain poultry production activities, such as commercial farming, increase the number of outbreaks at subdistrict level. It requires high poultry density and sustained host populations in order to increase the contact; therefore, high transmission rate to maintain the infectious chain and the persistence of HPAI-H5N1. It is evident that the prevalence of HPAI-H5N1 in the commercial poultry production system is high in Bangladesh [29], [41]. These results also support the results of previous ecological studies [4], [5], [11], [19]. Certain activities including management practices (e.g. level of biosecurity) in commercial poultry production, in Bangladesh, therefore, need to be revisited.

National highway networks crossing a subdistrict were found to increase the risk of HPAI-H5N1 outbreaks in the poultry population of that subdistrict. Previous studies confirmed the role of highway networks in the HPAI-H5N1 epidemiology of different countries [6], [11], [19], [42]. A national highway is a vital component of the poultry value chain as it is the main transportation route for poultry and poultry products. This poultry value chain component might also represent the main mechanism of spread of HPAI-H5N1 virus in Bangladesh. Transportation of infected poultry, contaminated poultry products, and equipment may be playing a role. The significance of highway networks as a risk factor warrants the importance of movement restrictions and monitoring the poultry movement within the country [6] to limit the magnitude of the epidemic.

Subdistricts with a high number of live bird markets had a higher risk of H5N1-HPAI outbreaks than subdistricts with a low number of live bird markets. Live bird markets are still popular and the main outlet for retail live birds in Bangladesh [12]. A live bird market could play a role as a dissemination point (hot spot) for H5N1-HPAI in Bangladesh, because live poultry from many different sources and geographical locations are brought there to be sold or slaughtered. Poultry is carried to market in metal cages or large bamboo cages. Both types of cages are often used for transport to and from markets without any cleaning/disinfection being carried out. Fecal materials piled up in the cages allow a long survival of the virus. Thus, movement of the cages themselves, to and from live bird markets may promote dissemination of the virus.

For the first time, in any ecological study for HPAI-H5N1, literacy rate was included in the analysis to adjust for potential reporting bias of the existing surveillance. As all other determinants in the final multivariable models were adjusted for literacy rate, the estimated associations could be interpreted as adjusted for possible surveillance bias. The current study demonstrated that subdistricts with a high literacy rate have a higher probability of reporting outbreaks. This higher literacy rate could probably be linked to awareness of the people regarding the disease and the compensation program that motivated them to report the suspected cases. We advocate more mass awareness programs and farmers education to facilitate early warning, rapid detection and quick response to HPAI-H5N1 outbreaks in Bangladesh; where the surveillance system largely depends on the farmers' report of the high morbidity and mortality in their flocks to the veterinary authority.

Contrary to previous findings in south-east Asian countries, duck density and crop intensity in the analysis of the present study were not associated with risk of HPAI-H5N1 outbreak. The role of free grazing ducks and rice agriculture has been discussed intensively, and their interacted ecology appeared to have contributory roles to HPAI outbreaks elsewhere [4], [13], [16]. However, it is not surprising that duck and crop intensity do not play the same role in the HPAI-H5N1 epidemiology, in Bangladesh. Duck husbandry in Bangladesh differs from the practices used in Thailand and Vietnam. In Bangladesh, large or commercial duck flocks are reared in marshy areas [12], which remain submerged most of the time and thus do not allow a high poultry density. Rearing ducks is not integrated with rice agriculture in Bangladesh. Moreover, like Thailand and Vietnam, long distance transport of a large number of ducks is a rare practice in Bangladesh. Thus, introduction and spread of the virus to other subdistricts via movement of ducks is unlikely.

The outcome variable for the present study was outbreak status at subdistrict level. In most cases, non-infected subdistrict without any outbreak report was not confirmed non-infected by sampling and subsequent laboratory tests. Therefore, some subdistricts classified as non-infected could have been truly infected; thus, cause some misclassification bias. This misclassification of the infection status is independent of the exposure status of the subdistrict, and, thus non-differential in nature. Furthermore, such misclassification would bias the result of the risk factors analysis towards the null-effect [43], suggesting even stronger ecological relations than those reported.

There are some limitations in the data set. We are lacking data on density of poultry of different types. Thus, this study is unable to infer anything on association between the risk of HPAI-H5N1 at subdistrict level in Bangladesh and poultry type. This association was shown earlier in other countries like Thailand [5]. Besides, data on illegal trades across borders were not available and thus was not operationalized in the models. Though, legal international trade of poultry in Bangladesh is limited to the importation of day-old chicks to grand parents and parents stock, illegal cross border trade is not a rare possibility [12]. Thus, diffusion of the virus through this practice cannot be ruled out. Moreover, we used the economic centers as a proxy of the live bird market. However, empirical data on the number of live bird market per subdistrict could have provided more reliable estimates.

The final model(s) from the two modeling approaches were different (Table 3). The results indicate that the ML model absorbed some of the covariate effect of the ICAR model and attributed that variation to the spatial random effect. This shift of the effect is due to the neighbor structure implied in the two different models. However, both models produced very similar predictive maps and had almost similar discriminating power concerning infected and non-infected subdistricts. The models are based on nationwide data, and the determinants assessed in the present study were carefully chosen by considering their plausible role in HPAI epidemiology. Therefore, we believe the discriminatory powers of the models are robust. Both models are statistically valid and represent commonly accepted modeling approaches. From the understanding of the representation of the spatial structure and empirical knowledge, the ICAR model is a reasonable choice for ecological modeling, though the ML is parsimonious, as it uses the simple hierarchy of the administrative units and does not need a complex correlation structure like the ICAR model.

In conclusion, the present study revealed that the ecological determinants influencing the number of outbreaks at subdistrict level in Bangladesh have similarities with other south-east Asian countries like Thailand and Vietnam but with noticeable differences. These country specific differences indicate that Bangladesh might have a different profile and mechanism of HPAI-H5N1 outbreaks. A statistical risk model based on the second wave of outbreaks in Thailand and a set of five environmental key factors maintained predictive power when extrapolated to Vietnam [3]. The same study supports such extrapolation to other countries with similar agro-ecological conditions. However, we recommend careful considerations concerning generalization of ecological studies involving different countries. Modeling ecological determinants require a sound analytical approach and local knowledge of the ecological factors. Mainly because of poultry trading and husbandry practices could differ significantly between countries with similar agro-ecological conditions. Local knowledge such as trading and husbandry practice of poultry is of paramount importance as they defines the connectivity among ecological determinants and hence, the epidemiology.

## Supporting Information

Figure S1 Distribution of migratory birds' staging areas in Bangladesh.
(TIF)

Figure S2 Illustration of different neighborhood structure used in (a) ICAR and (b) ML models.
(TIF)
