# Supplementary figures and images for: Ecological Determinants of Highly Pathogenic Avian Influenza (H5N1) Outbreaks in Bangladesh
Source: PLoS One. 2012 Mar 21;7(3):e33938. doi: 10.1371/journal.pone.0033938 (PMC3309954; doi:10.1371/journal.pone.0033938)

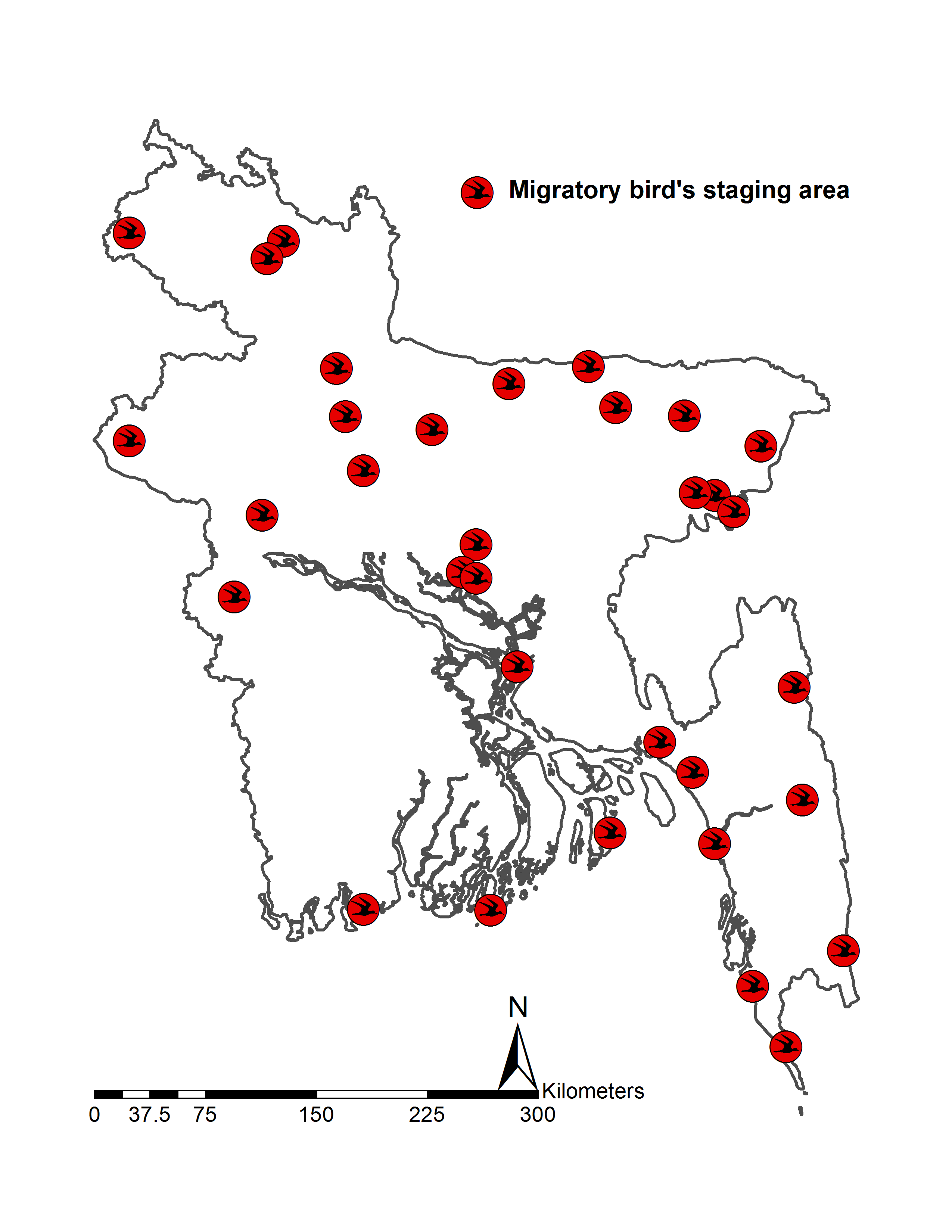

Supplement: Figure S1 — Distribution of migratory birds' staging areas in Bangladesh. (TIF) [file pone.0033938.s001.tif]

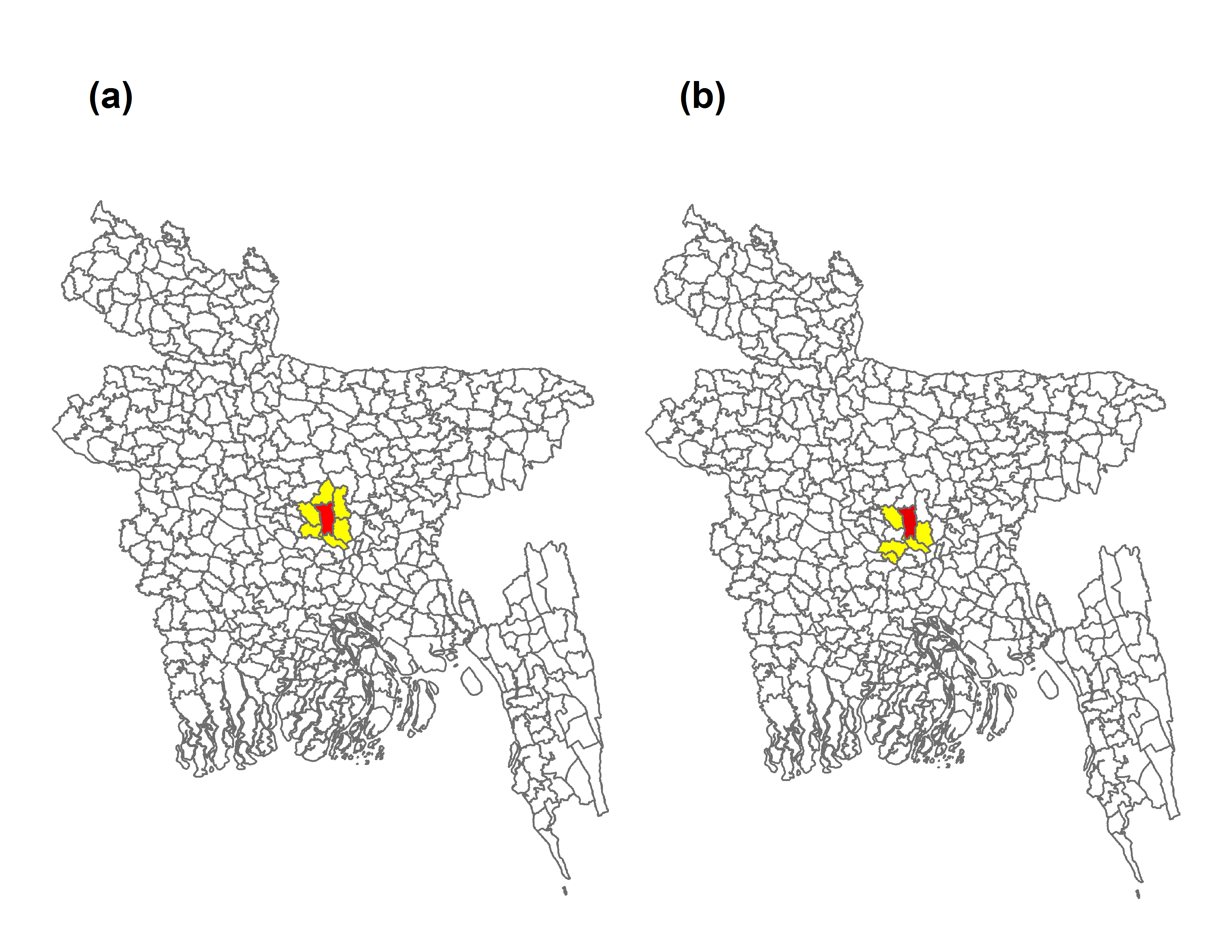

Supplement: Figure S2 — Illustration of different neighborhood structure used in (a) ICAR and (b) ML models. (TIF) [file pone.0033938.s002.tif]
